# Neurotoxicity and Intestinal Microbiota Dysbiosis in the Chinese Mitten Crab (*Eriocheir sinensis*) Induced by Anatoxin-a: A Microbiota–Intestine–Brain Axis Perspective

**DOI:** 10.3390/microorganisms13102380

**Published:** 2025-10-15

**Authors:** Huixia Feng, Shengyu Hu, Zihao Song, Ziqi Lin, Kai Zhang, Xianhui Ning, Cong Zhang, Shaowu Yin

**Affiliations:** 1 Jiangsu Province Engineering Research Center for Aquatic Animals Breeding and Green Efficient Aquacultural Technology, Jiangsu Key Laboratory of Ocean-Land Environmental Change and Ecological Construction, College of Marine Science and Engineering, Nanjing Normal University, Nanjing 210023, China; 2 Co-Innovation Center for Marine Bio-Industry Technology of Jiangsu Province, Lianyungang 222005, China

**Keywords:** *Eriocheir sinensis*, Anatoxin-a, neurotoxicity, microbiota dysbiosis, microbiota–intestine–brain axis

## Abstract

Anatoxin-a (ANTX-a), a potent neurotoxin produced by various cyanobacterial species, poses a serious threat to aquatic organisms. This study investigated the neurotoxicity of ANTX-a on juvenile Chinese mitten crab (*Eriocheir sinensis*). Different from previous studies on vertebrate models or fish liver toxicity, we focused on the microbiota–intestine–brain axis. Results demonstrated that ANTX-a exposure induced significant neurotoxicity, marked by the upregulation of apoptosis-related genes and disruption of neurotransmitter homeostasis. Transcriptomic analysis of thoracic ganglia revealed significant dysregulation of metabolic pathways, characterized by upregulated histidine metabolism (elevated histidine decarboxylase-like) and downregulated lipid metabolism (suppressed sphingomyelin phosphodiesterase-like). Additionally, increased intestinal histamine levels and elevated serum diamine oxidase activity indicated intestinal barrier damage. Intestinal microbiota analysis revealed that the abundance of nerve-related bacteria *Tyzzerella* and *Clostridium sensu stricto* 1 changed significantly. In summary, these findings indicate that ANTX-a induced neurotoxicity by affecting neurotransmitter systems and gut health, implicating the microbiota–intestine–brain axis. The results underscore the role of microbiota–intestine–brain communication in cyanotoxin toxicity within aquatic invertebrates and provide new insights into the ecotoxicological risks of cyanobacterial blooms in aquatic invertebrates.

## 1. Introduction

In recent years, due to the discharge of large amounts of chemical fertilizers and organic pollutants such as nitrogen-containing and phosphorus-containing wastewater into water bodies in human production and living activities, eutrophication of water bodies has become increasingly serious, and eutrophication of water bodies has exacerbated the frequent outbreak of cyanobacterial blooms. Some species of cyanobacterial blooms can produce cyanobacterial toxins, which can accumulate in aquatic biota and pose a great threat to the health and ecosystem of aquatic organisms [1]. Cyanotoxins are divided into four categories: neurotoxins, hepatotoxins, cytotoxins and stimulators, and gastrointestinal toxins [2]. Among the neurotoxins, anatoxins are neurotoxic toxins produced by *Anabaena flos-aquae* of Cyanobacterium [3]. Among them, ANTX-a is the most abundant toxin in the cells of cyanobacterial *Anabaena flos-aquae*, so it was first isolated and extracted [4].

The molecular structure of ANTX-a is very similar to that of the neurotransmitter acetylcholine, which can be used as an agonist of cholinergic receptors [5]. It will not be enzymatic hydrolysis, and thus high activity, toxicity, known as “very fast death factor” [6]. In fact, many acute animal poisoning incidents caused by ANTX-a have been reported around the world [7]. The acute effects of vertebrates mainly include a series of neurotoxic symptoms such as convulsions, muscle spasms, body imbalance, peripheral skeletal muscle and respiratory muscle paralysis, which can even lead to animal death symptoms after a few minutes [8,9]. ANTX-a is not very stable in aquatic environments because it degrades quickly under sunlight and high pH, but it tends to be enriched in aquatic organisms and causes potential food safety risks, such as *Cyprinus carpio*, *Pyrosomella verticilliata* and *Mytilus galloprovincialis* [10,11,12]. In addition, since *Anabaena flos-aquae* lives in the aquatic ecosystem, aquatic organisms have also become the first victims of ANTX-a. At present, the research on ANTX-a on aquatic animals is mainly concentrated on fish. For example, *Oryzias latipes* will be suffocated and die of respiratory paralysis in 1~2 min under the condition of 20 μg·L^−1^ ANTX-a [13]. ANTX-a exposure can also cause immunotoxicity of *Carassius auratus*, such as decreased activity of immune cells (neutrophils, lymphocytes, macrophages and bone marrow cells) and immune system disorders [14]. In *C. auratus*, studies also have found that ANTX-a can lead to lymphocyte nuclear chromatin condensation, vacuolization and mitochondrial swelling [7]. However, the current reports on the neurotoxicity of ANTX-a are mostly focused on vertebrates and rarely report on aquatic invertebrates.

To broaden our understanding of ANTX-a’s neurotoxicity, its effects must be evaluated across a wider range of aquatic species. Crustaceans, a major invertebrate lineage, are particularly relevant models due to their comparatively well-developed central nervous system (CNS) [15]. Among them, the Chinese mitten crab, *Eriocheir sinensis*, serves as a key indicator species for assessing aquatic contamination [16], which belongs to crustaceans and usually prefers to live in rivers and lakes with clean water quality and abundant aquatic plants. In addition, previous studies have found that *Aphanizomenon* sp. isolated from Tien Lake in China has the ability to produce ANTX-a [3]. Crabs are omnivorous animals, which greatly increases the risk of ANTX-a poisoning in *E. sinensis*.

Furthermore, while *E. sinensis* lacks a complex, vertebrate-like brain, it possesses a differentiated CNS consisting of cranial and thoracic ganglia. Investigating the specific function of this CNS during the crab’s response to ANTX-a-induced stress is an area that warrants deeper exploration. Consequently, the present study utilized the Chinese mitten crab as a representative invertebrate model to investigate the CNS response to ANTX-a. Our primary objectives were twofold: first, to clarify the mechanisms by which ANTX-a exerts neurotoxic effects in juvenile *E. sinensis*, and second, to probe the involvement of the microbiota-gut–brain axis in this toxicological process. The findings contribute to a more profound comprehension of ANTX-a’s neurotoxic action in aquatic fauna and offer new perspectives on the ecological risks associated with its presence.

## 2. Materials and Methods

### 2.1. Animals and Chemicals

Juvenile *E. sinensis* were purchased from a commercial farm site in Chongming, Shanghai, and were acclimatize to the laboratory conditions (temperature: 23.5 °C ± 1 °C, pH: 7.2–7.6, dissolved oxygen: 6.8 ± 0.2 mg/L) for two weeks. Crabs were reared in tanks. ANTX-a used in this test was purchased from GLPBIO (Cas No.:1219922-30-1, Montclair, CA, USA).

### 2.2. Experimental Design

Following an acclimation period, 360 healthy juvenile crabs, with an average weight of 6.5 ± 0.5 g, were randomly allocated into six experimental groups. Each group consisted of five replicate tanks, with 12 crabs in each tank. For dosing, a stock solution of ANTX-a fumarate was formulated in phosphate-buffered saline (PBS, pH 7.4) at a concentration of 1 mg/mL. By properly diluting the stock solution with PBS, injection solutions of desired concentration of ANTX-a (0.16, 0.32, 0.64, 1.28 and 2.56 μg of ANTX-a g^−1^ crab weight; bw) were freshly obtained. In the pre-experiment, we determined the formal test concentration by injecting 2 μL per gram of bw of several doses of ANTX-a. Within 10 min, individuals taking 2.56 μg of ANTX-a g^−1^ of crab bw had tight limbs and could not move. After 10 min, the limbs gradually stretched and died. Therefore, the dose of 2.56 μg ANTX-a g^−1^ bw was selected as the highest concentration value in the formal test for definitive determination, and other concentration values were set according to the ratio.

Crabs in the treatment groups were injected with different concentrations of ANTX-a, and the control group was injected with PBS solution. After injection, the crabs were quickly transferred to the water tank. The samples were collected for parameter analysis after the experiment lasted for 96 h.

### 2.3. Biochemical Assay

To assess intestinal barrier integrity, six crabs were selected from each group to measured serum diamine oxidase (DAO) activity and intestinal histamine (HIS) concentrations using enzyme-linked immunosorbent assay (ELISA) kits obtained from Jiangsu Meimian Industrial Co., Ltd. (Yancheng, China) as per the supplier’s instructions.

### 2.4. Quantitative Real-Time PCR

For gene expression assessment, six thoracic ganglia samples were randomly chosen from every group. The process involved total RNA isolation, verification of its integrity, and subsequent reverse transcription. The synthesized cDNA was then used for qRT-PCR, following the detailed methodology described by [15]. All primer sequences are listed in Table S1, and the 2^−ΔΔCt^ method was employed to calculate relative mRNA abundance.

### 2.5. Transcriptome Sequencing (RNA-Seq) Analysis

RNA-Seq analysis was conducted on the Control, 0.32, and 1.28 µg/L groups, as these showed notable physiological changes. And five juvenile crabs were taken from each group for RNA-Seq analysis. Total RNA from thoracic ganglia was isolated with TRIzol reagent (Invitrogen Life Technologies, Carlsbad, CA, USA). A NanoDrop spectrophotometer (Thermo Scientific, Waltham, MA, USA) was used to evaluate RNA concentration and purity. For validation of the sequencing data, six genes were selected at random for quantification by qRT-PCR. The bioinformatics pipeline and validation procedures were carried out according to previously established methods [15].

### 2.6. Microbiome Profiling

In order to examine the changes in gut microbiota caused by ANTX-a, we extracted total bacterial genomic DNA from the intestinal contents of five crabs in Control, 0.32 and 1.28 µg/g groups. This was done using a commercial kit, strictly adhering to the manufacturer’s guidelines. The purity and concentration of the extracted DNA were assessed using a NanoDrop 2000 spectrophotometer (Thermo Scientific, Waltham, MA, USA) and through agarose gel electrophoresis. A full description of the microbiome analysis can be found in the Supplementary Methods.

### 2.7. Integrative Analysis of Intestinal Microbiome and Metabolomics

Hierarchical clustering and Pearson correlation algorithms were applied to identify associations between intestinal microbial communities and host transcriptomic profiles in *E. sinensis*. Heatmap visualization was performed to delineate co-occurrence patterns.

### 2.8. Statistical Analyses

Statistical significance was determined using one-way ANOVA followed by Duncan’s test in SPSS Version 22.0 (IBM Corp., Armonk, NY, USA). All data are reported as the mean ± standard error (SE), with *p* < 0.05 considered statistically significant. Prior to analysis, all datasets were assessed for normality (Shapiro–Wilk test) and homoscedasticity (Levene’s test).

## 3. Results

### 3.1. Apoptotic Analysis

**Figure 1:**
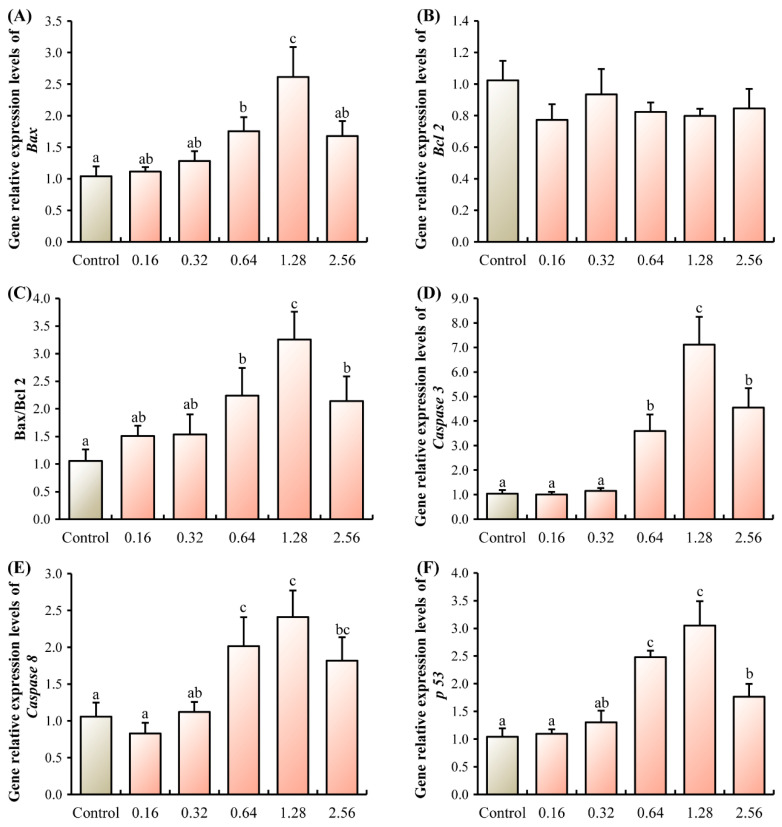
Effects of ANTX-a on the mRNA expression levels of apoptosis-related genes of juvenile *E. sinensis.* (**A**) *Bax*, Bcl-2 associated X protein; (**B**) *Bcl-2*, B-cell lymphoma-2; (**C**) Ratio of Bax to Bcl2; (**D**) *Caspase 3*, cysteinyl aspartate specific proteinase 3; (**E**) *Caspase 8*, cysteinyl aspartate specific proteinase 8; (**F**) *p53*, tumor Protein p53. Different letters shown above the columns represent significant differences (*p* < 0.05).

ANTX-a exposure triggered a significant upregulation in the mRNA level of the pro-apoptotic gene *Bax* at concentrations of 0.64 and 1.28 µg/L relative to the control group (*p* < 0.05, Figure 1A). A similar increasing trend was observed for the Bax/Bcl-2 ratio and the transcript levels of *Caspase 3*, *Caspase 8*, and *p53* in all treatment groups from 0.64 to 2.56 µg/L. These indicators peaked at the 1.28 µg/L exposure level, showing a marked elevation compared to controls (*p* < 0.05, Figure 1C–F).

### 3.2. Neurotransmitter Homeostasis

**Figure 2:**
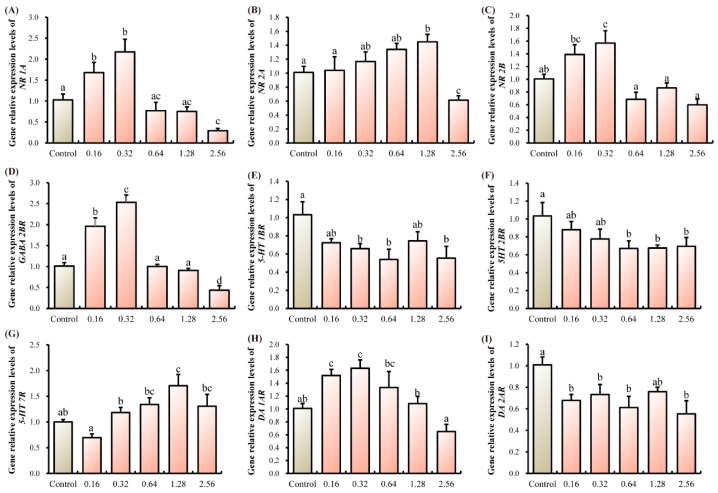
Effects of ANTX-a on the mRNA expression levels of neurotransmitter receptor-related genes of juvenile *E. sinensis*. (**A**–**C**) *NR*, N-methyl-D-aspartate receptor; (**D**) *GABA 2BR*, γ-aminobutyric acid type B receptor subunit 2; (**E**–**G**), *5-HT*, serotonin receptor; (**H**,**I**) DA, dopamine receptor. Different letters shown above the columns represent significant differences (*p* < 0.05).

Within the thoracic ganglia, transcript levels of NMDA receptors (*NR 1A*, *NR 2A*, *NR 2B*) and *GABA 2BR* initially rose before declining as ANTX-a concentration increased. Specifically, *NR 1A*, *NR 2B*, and *GABA2BR* expression peaked in the 0.32 µg/L group, whereas *NR 2A* expression was highest in the 1.28 µg/L group (*p* < 0.05, Figure 2A–D). For serotonin receptors, transcript levels of *5-HT 1BR* and *5-HT 2BR* were significantly reduced in the 2.56 µg/L group compared to controls, while *5-HT 7R* expression peaked at the 1.28 µg/L dose (*p* < 0.05, Figure 2E–G). Regarding dopamine receptors, *DA 1AR* transcripts were significantly elevated in the 0.16 and 0.32 µg/L groups, whereas an inverse trend was observed for *DA 2AR* (*p* < 0.05, Figure 2H,I).

### 3.3. Intestinal Injury Indicators

**Figure 3:**
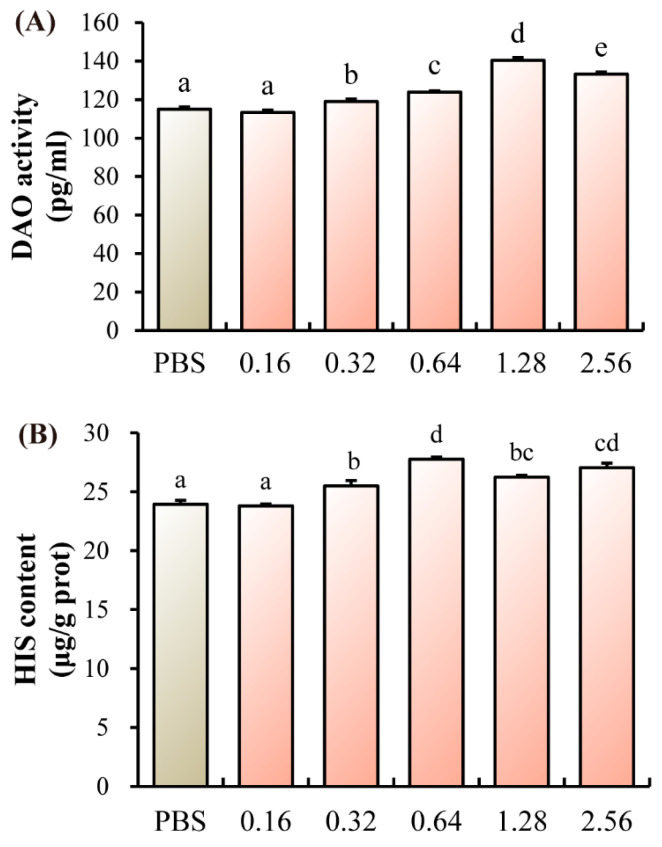
Effects of ANTX-a on intestinal barrier integrity of juvenile *E. sinensis*. (**A**) Serum diamine oxidase activity; (**B**) Intestinal histamine concentration. Different letters shown above the columns represent significant differences (*p* < 0.05).

Relative to the control group, a significant elevation in both DAO activity and HIS content was observed in all treatment groups ranging from 0.32 to 2.56 µg/L (*p* < 0.05, Figure 3A,B).

### 3.4. Intestinal Flora Analysis

#### 3.4.1. Intestinal Microbiota Diversity Analysis

A Venn diagram analysis revealed a core of 423 OTUs shared among the Control, 0.32, and 1.28 µg/L groups. The number of OTUs unique to each group was 37 for the Control, 8 for the 0.32 µg/L group, and 77 for the 1.28 µg/L group (Figure S1A,B). The index of Chao 1, Simpson, ACE and PD showed an upward trend with ANTX-a treatment (Figure S1C–F). In addition, the beta diversity analysis by PCA showed that the Control group and the stress groups formed different clusters, and the first two principal components (PCA1 and PCA2) explained 39.77% and 16.69% of the variation, respectively (Figure S1G). Similarly, the results of PCoA analysis of juvenile crab under the stress of ANTX-a showed that different intestinal microorganisms were induced at the family level, and the separation was obvious (Figure S1H). The PCoA showed that PCoA1 and PCoA2 explained 40.08% and 18.43% of the variation, respectively.

#### 3.4.2. Microbial Species Analysis

**Figure 4:**
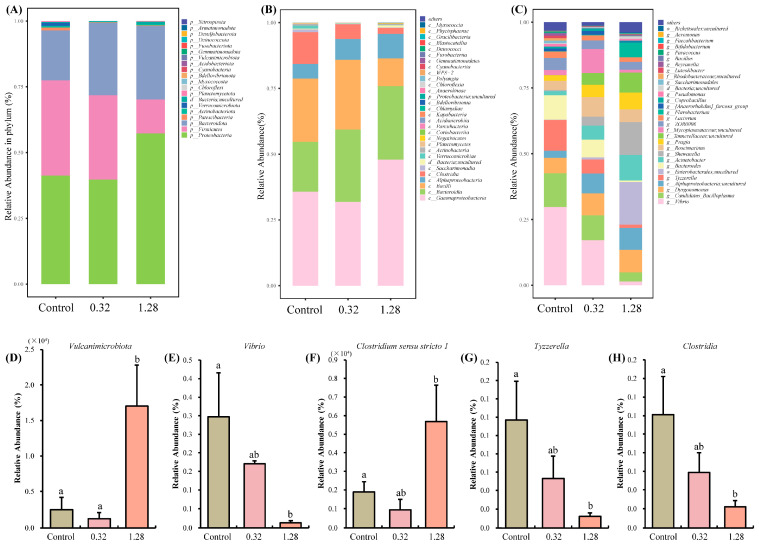
The intestinal microbiota composition and diversity in different groups of the juvenile *E. sinensis*. (**A**) Phylum composition; (**B**) Class composition; (**C**) Genus composition; (**D**) Comparison of relative abundance of *Vulcanimicrobiota*; (**E**) Comparison of relative abundance of *Vibrio*; (**F**) Comparison of relative abundance of *Clostridium sensu stricto 1*; (**G**) Comparison of relative abundance of *Tyzzerella*; (**H**) Comparison of relative abundance of *Clostridia*. Different letters represent significant differences (*p* < 0.05) among groups.

Analysis of the microbial composition at the phylum level indicated that Proteobacteria, Firmicutes, and Bacteroidetes were the predominant phyla across the Control, 0.32, and 1.28 µg/L groups (Figure 4A). Notably, the relative abundance of Vulcanimicrobiota was significantly elevated in the 1.28 µg/L group compared to both the control and 0.32 µg/L groups (*p* < 0.05, Figure 4D). At the class level, Gammaproteobacteria, Bacteroidia, and Bacilli were the most common classes (Figure 4B), while the abundance of Clostridia was significantly depleted in the 1.28 µg/L group relative to the control (*p* < 0.05, Figure 4H). At the genus level, *Enterobacterales*, *Shewanella*, and *Acinetobacter* showed increased abundance and became dominant in the 1.28 µg/L group (Figure 4C). One-way ANOVA identified a significant reduction in the abundance of both *Vibrio* and *Tyzzerella* (*p* < 0.05, Figure 4E,G). Of particular note, the abundance of *Clostridium sensu stricto 1* was significantly lower in the 1.28 µg/L group than in the control group (*p* < 0.05, Figure 4F).

### 3.5. Transcriptomic Alterations

#### 3.5.1. Transcriptome Sequence Evaluation and Annotation

Thoracic ganglia from three groups of juvenile *E. sinensis* were subjected to RNA sequencing. This generated a total of 628,935,096 raw reads, which yielded 628,481,522 clean reads following quality control and data filtering. The GC content for all samples exceeded 36.55%. Furthermore, the Q20 and Q30 scores for each sample were consistently above 98.43% and 95.28%, respectively (Table S2), confirming that the sequencing data was of high quality suitable for downstream analyses.

#### 3.5.2. Novel Gene Prediction

**Figure 5:**
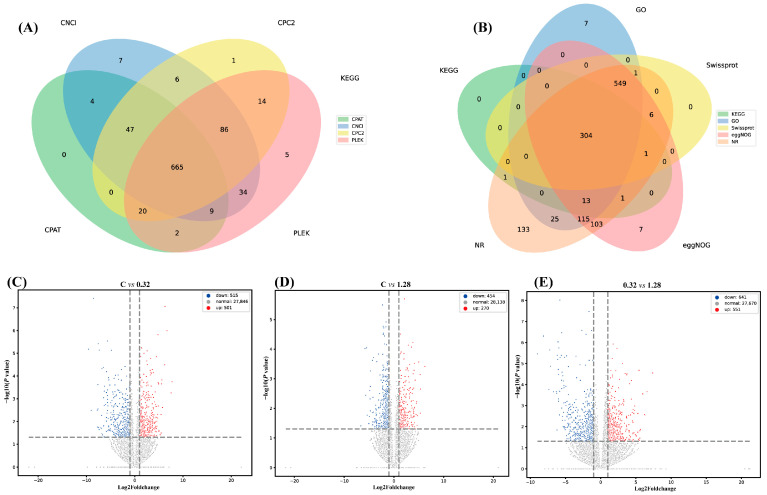
Effects of ANTX-a on thoracic ganglia transcriptome different-expressed genes (DEGs) of juvenile *E. sinensis*. (**A**) Venn diagram of coding potential prediction by four tools (CNCI, CPC2, CPAT, PLEK); (**B**) Venn diagram of functional annotation of novel transcripts against five databases (NR, KEGG, GO, eggNOG, SwissProt); (**C**) Volcano plot showing DEGs in C group vs. 0.32 group; (**D**) Volcano plot showing DEGs in C group vs. 1.28 group; (**E**) Volcano plot showing DEGs in 0.32 group vs. 1.28 group.

The Venn diagram results showed that the number of genes shared by the four groups of CNCI, CPC2, CPAT and PLEK was 665, of which the number of genes unique to CNCI, CPC2, CPAT and PLEK was 7, 1, 0 and 5, respectively (Figure 5A). In addition, the results showed that the number of genes shared by NR, KEGG, GO, eggNOG and Swissprot databases was 304, of which the number of genes in NR database was 1252, the number of genes in KEGG database was 320, the number of genes in GO database was 1014, the number of genes in eggNOG database was 1099, and the number of genes in Swissprot database was 861 (Figure 5B).

#### 3.5.3. Differentially Expressed Gene Analysis

Pairwise comparisons between the three sequencing libraries identified the differentially expressed genes (DEGs) (Table 1 and Table 2). In the 0.32 µg/L group versus the Control, 501 genes were found to be upregulated and 515 were downregulated (Figure 5C). When comparing the 1.28 µg/L group to the Control, 270 genes were upregulated while 454 were downregulated (Figure 5D). Volcano plots revealed that in both treatment groups, the number of downregulated DEGs exceeded the number of upregulated DEGs. Furthermore, the comparison between the 0.32 µg/L and 1.28 µg/L groups identified 551 upregulated and 641 downregulated genes.

#### 3.5.4. Functional Enrichment Analysis of Differentially Expressed Genes

**Figure 6:**
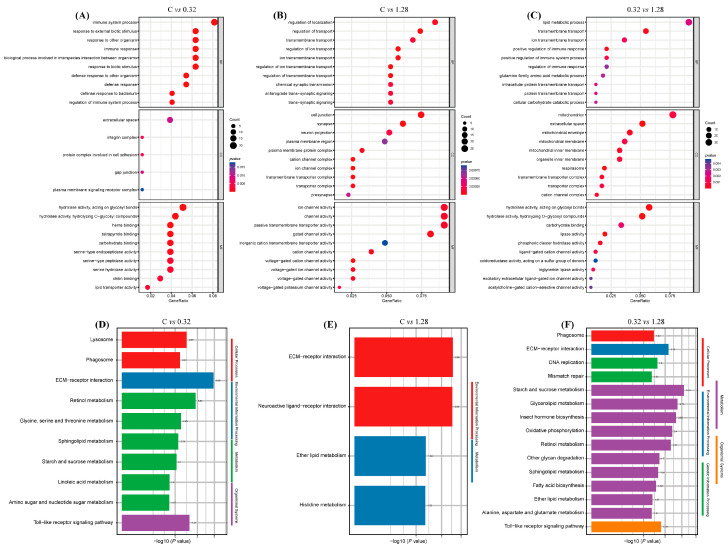
Effects of ANTX-a on thoracic ganglia transcriptome pathways of juvenile *E. sinensis*. (**A**) GO enrichment analysis of C group vs. 0.32 group; (**B**) GO enrichment analysis of C group vs. 1.28 group; (**C**) GO enrichment analysis of 0.32 group vs. 1.28 group; (**D**) KEGG enrichment analysis of C group vs. 0.32 group; (**E**) KEGG enrichment analysis of C group vs. 1.28 group; (**F**) KEGG enrichment analysis of 0.32 group vs. 1.28 group.

To further investigate the global impact of ANTX-a on the physiological functions of juvenile crabs, we performed functional annotation of the DEGs using the Gene Ontology (GO) and Kyoto Encyclopedia of Genes and Genomes (KEGG) databases. Significantly enriched GO terms were categorized into biological process (BP), cellular component (CC), and molecular function (MF). For the C vs. 0.32 µg/L comparison, the most enriched BP terms were related to ‘response to external biotic stimulus’ and ‘immune response’. Within the CC category, ‘integrin complex’ and ‘protein complex involved in cell adhesion’ were the top terms. For MF, ‘hydrolase activity, hydrolyzing O-glycosyl compounds’ and ‘hydrolase activity, acting on glycosyl bonds’ were the two most significantly enriched functions (Figure 6A).

The pathways significantly enriched in biological processes in C group vs. 1.28 group were mainly regulation of ion transport and chemical synaptic transmission. The cation channel complex and synapse were the first two most significantly enriched in the cellular components. Among the molecular functions, voltage-gated cation channel activity was the most significant (Figure 6B). The pathways significantly enriched in biological processes in 0.32 group vs. 1.28 group were mainly transmembrane transport and positive regulation of immune response. The top two cell components were extracellular space and respirasome. Hydrolase activity, hydrolyzing O-glycosyl compounds and hydrolase activity, acting on glycosyl bonds were the top two molecular functions with the most significant enrichment (Figure 6C).

KEGG enrichment analysis for the C vs. 0.32 µg/L comparison identified 10 significantly altered terms, falling into four main categories: Environmental Information Processing, Metabolism, Organismal Systems, and Cellular Processes. Metabolic pathways were highly represented, including Sphingolipid metabolism, Starch and sucrose metabolism, and Retinol metabolism. ‘ECM-receptor interaction’ was the predominant pathway in Environmental Information Processing, while ‘Lysosome’ and ‘Phagosome’ were the main pathways in Cellular Processes. The ‘Toll-like receptor signaling pathway’ was the primary affected pathway in Organismal Systems (Figure 6D). In the C vs. 1.28 µg/L comparison, Environmental Information Processing was a dominant category, featuring ‘ECM-receptor interaction’ and ‘Neuroactive ligand-receptor interaction’. Enriched metabolic pathways included ‘Ether lipid metabolism’ and ‘Histidine metabolism’ (Figure 6E). Finally, the comparison between the 0.32 and 1.28 µg/L groups yielded 15 significantly altered KEGG terms across five categories, with metabolic pathways again constituting a major portion (Figure 6F).

#### 3.5.5. RNA-Seq Validation by qRT-PCR

To confirm the validity of the RNA-Seq data, we performed qRT-PCR on six selected DEGs: *AKT*, *Bcl-2*, phenoloxidase (*PO*), catalase (*CAT*), Cu-Zn Superoxide Dismutase (*Cu-ZnSOD*), and Toll-like receptor (*TLR*). The expression patterns of these genes determined by qRT-PCR were in agreement with the RNA-Seq findings (Figure S2), thereby supporting the reliability of our transcriptomic results.

### 3.6. Comprehensive Flora and Transcriptome Analysis

**Figure 7:**
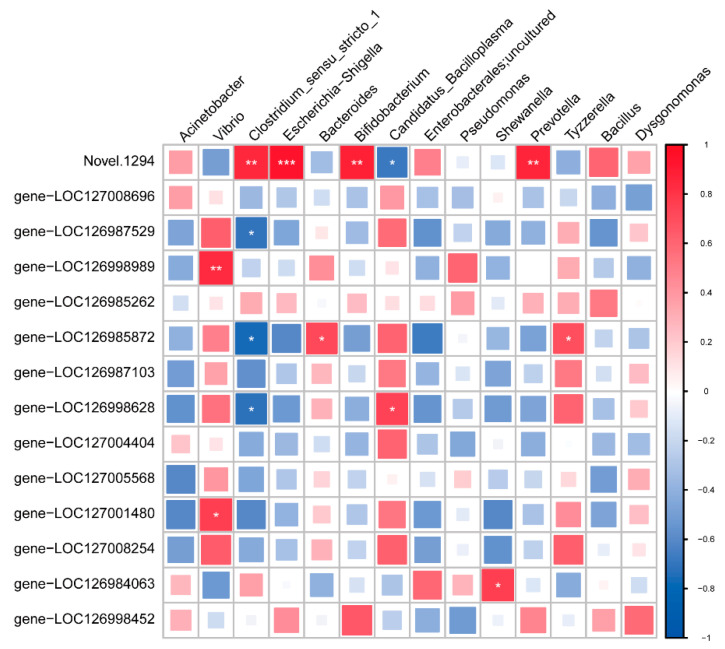
Integrative analysis of the correlation between microbiome and transcriptome in juvenile E. sinensis. The heatmap displays Pearson correlation coefficients. Red indicates a positive correlation, and blue indicates a negative correlation. Asterisks (*), (**), (***) denotes a statistically significant correlation *p* < 0.05, *p* < 0.01 and *p* < 0.001, respectively.

A correlation analysis was conducted between genus-level gut bacteria and thoracic ganglion gene expression. This revealed a positive association between the abundance of *Vibrio* and the transcript levels of gene-LOC126998989 (gamma-aminobutyric acid receptor alpha-like) and gene-LOC127001480 (glutamate receptor ionotropic, NMDA 2B-like). In addition, *Shewanella* was well correlated with gene-LOC126984063 (D(2) dopamine receptor-like). *Clostridium sensu stricto 1* was negatively related to gene-LOC126987529 (gamma-aminobutyric acid receptor subunit alpha-6-like) (*p* < 0.05, Figure 7).

## 4. Discussion

Consistent with its known role as a potent neurotoxin, our study demonstrates that ANTX-a induces significant neurotoxicity in the juvenile *E. sinensis*. Building on previous research in vertebrates, we provide novel evidence from an invertebrate model, implicating the microbiota–intestine–brain axis in this toxicological process. The observed alterations in the expression of apoptosis-related genes in the thoracic ganglia indicates that ANTX-a triggers neuronal apoptosis in crabs, potentially through both intrinsic and extrinsic pathways [17,18]. This aligns with findings in carp lymphocytes, where ANTX-a also acts as a potent apoptotic inducer [7,10].

It is interesting that we monitored the behavior of crabs for the first 10 min after injection of ANTX-a. The acute neurotoxic effect was further evidenced by behavioral deficits, including limb curling, loss of balance, and did not respond to touch, following high-dose ANTX-a injection. Behaviorally, the crabs’ cheliped began to stretch gradually after one hour of exposure. At the cellular level, neuronal communication relies on neurotransmitters, which are synthesized from precursors and liberated into the synaptic cleft to bind with their respective receptors on presynaptic or postsynaptic membranes. Following signaling, these neurotransmitters are cleared from the synapse via reuptake by presynaptic transporters and are subsequently broken down by specific enzymes [19,20]. ANTX-a, as a competitive agonist of acetylcholine, binds to its specific membrane receptor (mAChR), which cannot be degraded by acetylcholinesterase, resulting in blocked neuromuscular signal transmission, excessive stimulation of muscle cells and continuous contraction [13]. Behavioral deficits in fish such as *C. carpio* and *C. auratus*, such as rapid eye movement, abnormal swimming and muscle stiffness, have also been observed in some experiments [4,21]. Previous studies have shown that below a certain level, this effect seems to be transient, with animals able to recover completely quickly, although the data available in the literature is still limited [8].

Effective neural conduction hinges on maintaining a precise equilibrium between excitatory and inhibitory neurotransmission [22]. Our data reveal that ANTX-a disrupted this balance, as shown by the dysregulated expression of receptors for key neurotransmitters (NMDA, DA, 5-HT, GABA) in the thoracic ganglia. The NMDA receptor (NMDA-R) is a key ionotropic glutamate receptor that functions as a ligand- and voltage-gated channel. It is activated by the neurotransmitter glutamate and is characterized by its high permeability to both calcium and sodium ions [15]. *DA* is one of the most important catecholamine neurotransmitters in the CNS, which plays an important role in regulating the movement, cognition, emotion, positive reinforcement, feeding and endocrine of the brain [23]. Studies have shown that the *DA* regulation of intracellular cascades also enhances *NMDA*-induced responses [24]. Serotonin (5-HT), an inhibitory neurotransmitter primarily produced by enterochromaffin cells, serves a critical function in processes such as neural development and the formation of synapses [25]. Some studies have shown that *5-HT* neurons in the CNS can affect many other neurons and regulate their neurotransmitter release [26]. *GABA* is a major inhibitory neurotransmitter in the CNS, *GABA-R* widely distributed in neurons and glial cells and play an important role in regulating neurotransmitter release and neuronal excitability [27,28]. In this study, the neurotransmitter signaling pathway composed of *5-HT*, *DA*, *NMDA* and *GABA* signaling pathways was affected by ANTX-a, which was manifested as the instability of neurotransmitter receptor expression level.

In addition, our findings indicate that ANTX-a exposure led to substantial alterations in the transcriptomic profile of the thoracic ganglia in juvenile crabs. In both the 0.32 µg/L and 1.28 µg/L treatment groups, the number of downregulated DEGs was greater than that of upregulated DEGs when compared to controls, a trend that was particularly pronounced in the 1.28 µg/L group. This suggests that ANTX-a might disrupt the fundamental physiological processes of *E. sinensis* through widespread gene expression inhibition, thereby compromising their overall health and survival. Similar results were found in previous studies on the hepatotoxicity of acute GLA exposure to juvenile crabs [29]. KEGG pathway enrichment provided supporting evidence for the neurotoxicity of ANTX-a.

Among them, the co-enriched pathways of C group vs. 0.32 group and C group vs. 1.28 group had ECM-receptor interaction, which is crucial for neuronal plasticity axonal regeneration [30]. We speculated that juvenile crabs start self-protection mechanisms to accelerate nerve cell activity after ANTX-a stress. Moreover, ANTX-a also had a significant effect on lipid metabolism pathways such as Sphingolipid metabolism, Linoleic acid metabolism and Ether lipid metabolism in the thoracic ganglia. Among them, sphingolipids and their enzyme systems contain multiple and interrelated signaling pathways in the CNS, coordinate CNS physiological processes, and participate in excessive neuroinflammation and neurodegenerative diseases [31]. Its related DEGs were also significantly up-regulated. In particular, the 1.28 group of juvenile crabs also initiated the Histidine metabolic pathway. Histidine is a bioactive amino acid in the nervous system and a precursor of histamine (HA) [32]. In addition, in the Neuroactive ligand-receptor interaction pathway of the 1.28 group, we found some neurotransmitter receptor genes that are also significantly expressed in qRT-PCR, such as *5-HT R* and *NMDA 2BR*. Generally, ANTX-a can lead to significant changes in the transcriptome of the thoracic ganglia of *E. sinensis*, which may mainly cause toxic damage to the nerves of juvenile crabs by mediating ECM-receptor interaction, Lipid metabolism, Histidine metabolism and Neuroactive ligand-receptor interaction pathways.

A growing body of evidence highlights the crucial role of the gut microbiota in host physiology. These commensal microorganisms can synthesize a diverse array of neuroactive molecules, including neurotransmitters, hormones, and short-chain fatty acids (SCFAs) [33,34]. Such microbial-derived signals are capable of entering the systemic circulation, crossing the blood–brain barrier, and ultimately modulating neural function and behavior [35]. For example, chronic treatment with Lactobacillus rhamnosus (JB-1) in mice induced a region-dependent change in GABA_B1b_ mRNA in the brain, and JB-1 reduced stress-induced corticosterone and anxiety and depression-related behaviors [36]. However, due to the lack of protective mechanisms such as blood–brain barrier, ANTX-a is likely to cause damage to its nerves through intestinal or flora metabolites. In our study, the significant increase in DAO activity and HIS content indicated that ANTX-a caused intestinal damage in juvenile crabs. Moreover, the intestinal flora of juvenile crabs was disordered after ANTX-a injection. Especially at the Genus level, the richness of *Tyzzerella* in 1.28 group was significantly lower than that in the Control group. *Tyzzerella* is a member of the Lachnospiraceae family, which is one of the symbiotic bacteria in the human gut microbiota [37,38]. For example, *Tyzzerella nexilis*, whose abundance is significantly associated with neurological dysfunction [39]. Furthermore, we observed a significant increase in the abundance of *Clostridium sensu stricto 1* in the 1.28 µg/L group. Members of the *Clostridium* genus are recognized as major producers of SCFAs and play key regulatory roles in synaptic transmission, neurotransmitter synthesis, and immune and inflammatory signaling [40]. Interestingly, the abundance of the class Clostridia in the 1.28 µg/L group showed an opposite trend to that of the genus *Clostridium sensu stricto 1*, a discrepancy that might reflect nervous system dysregulation in the crabs. ANTX-a clearly triggered significant shifts in the gut microbial community of juvenile crabs. This dysbiosis could indirectly alter neurotransmitter release, thereby disrupting the homeostasis of the neurotransmitter system and leading to neurological impairment. These observations align with a prior study on glufosinate-ammonium (GLA) toxicity, which also reported concurrent gut dysbiosis and neurotoxicity in juvenile *E. sinensis* [41]. Taken together with our correlation analysis, these findings underscore the intimate link between the gut microbiome and the neural transcriptome, suggesting an interaction between these physiological systems and offering a new avenue for investigating the neurotoxic mechanisms of ANTX-a.

## 5. Conclusions

In summary, this study provides evidence that ANTX-a exerts neurotoxic effects in *Eriocheir sinensis* through the induction of neuronal apoptosis and dysregulation of neurotransmitter systems. More importantly, we reveal the crucial involvement of the microbiota–intestine–brain axis in this process. ANTX-a not only causes intestinal barrier dysfunction but it triggers significant shifts in gut microbial composition, particularly in bacteria associated with neural function. The concomitant transcriptomic changes in ganglia support a model of toxin-induced disruption of microbiota–intestine–brain communication. These findings underscore direct ecological risks. The neurobehavioral and intestinal damage we observed suggests that ANTX-a contamination could compromise key fitness traits in wild crabs, including foraging efficiency and predator avoidance, thereby affecting natural population dynamics and causing cascading effects throughout aquatic food webs. From an applied perspective, these results also highlight a serious threat to aquaculture safety and productivity, emphasizing the need to monitor cyanobacterial toxins for the protection of both aquatic ecosystems and sustainable aquaculture industries.

## Figures and Tables

**Table 1:** Selected differentially expressed genes (DEGs) in the thoracic ganglia of juvenile *E. sinensis* from the comparison between the Control and 0.32 µg/g ANTX-a groups.

Gene ID	Gene Description	Pathway	Control vs. 0.32
gene-LOC127004233	cell adhesion molecule DSCAML1-like	ECM-receptor interaction	up
gene-LOC127005077	agrin-like	ECM-receptor interaction	up
gene-LOC127006442	integrin alpha-PS2-like	ECM-receptor interaction	up
gene-LOC126981348	oplophorus-luciferin 2-monooxygenase non-catalytic subunit-like	ECM-receptor interaction	up
gene-LOC126988411	integrin alpha-PS3-like	ECM-receptor interaction	up
gene-LOC127009668	sphingomyelin phosphodiesterase-like	Sphingolipid metabolism	up
gene-LOC126997840	putative glucosylceramidase 4	Sphingolipid metabolism	up
gene-LOC126983484	arylsulfatase A-like	Sphingolipid metabolism	up
gene-LOC126985871	sphingosine-1-phosphate phosphatase 2-like	Sphingolipid metabolism	up

**Table 2:** Selected differentially expressed genes (DEGs) in the thoracic ganglia of juvenile *E. sinensis* from the comparison between the Control and 1.28 µg/g ANTX-a groups.

Gene ID	Gene Description	Pathway	Control vs. 1.28
gene-LOC127006442	integrin alpha-PS2-like	ECM-receptor interaction	up
gene-LOC126988417	integrin alpha-PS3-like	ECM-receptor interaction	up
gene-LOC127000170	ficolin-1-like	ECM-receptor interaction	down
gene-LOC127008206	glutamate receptor ionotropic, kainate 4-like	Neuroactive ligand-receptor interaction	up
gene-LOC127005040	5-hydroxytryptamine receptor-like	Neuroactive ligand-receptor interaction	down
gene-LOC126981054	diuretic hormone receptor-like	Neuroactive ligand-receptor interaction	down
gene-LOC126983114	muscarinic acetylcholine receptor DM1-like	Neuroactive ligand-receptor interaction	down
gene-LOC126983773	galanin receptor 2a-like	Neuroactive ligand-receptor interaction	down
gene-LOC126986214	glutamate receptor ionotropic, NMDA 2B-like	Neuroactive ligand-receptor interaction	down
gene-LOC127003406	histidine decarboxylase-like	Histidine metabolism	down

## Data Availability

Data is contained within the article or Supplementary Material. The original contributions presented in this study are included in the article/Supplementary Material. Further inquiries can be directed to the corresponding authors.

## Supplementary Materials

The following supporting information can be downloaded at: https://www.mdpi.com/article/10.3390/microorganisms13102380/s1, Figure S1: The diversity of intestinal microbial in different groups of the juvenile *E. sinensis*; Figure S2: Validation of gene expression patterns by qPCR; Table S1: Primer information for quantitative real-time polymerase chain reaction; Table S2: Evaluation of RNA-Seq Data.

## References

[1] Ibelings B.W., Havens K.E. (2008). Cyanobacterial toxins: A qualitative meta-analysis of concentrations, dosage and effects in freshwater, estuarine and marine biota. Adv. Exp. Med. Biol..

[2] Codd G.A., Morrison L.F., Metcalf J.S. (2005). Cyanobacterial toxins: Risk management for health protection. Toxicol. Appl. Pharmacol..

[3] Luo C., Wang S., Zuo J., Luo Y., Gan N. (2022). Research and Prospects of Eco-toxicity of Anatoxins. Asian J. Ecotoxicol..

[4] Carmichael W.W., Biggs D.F., Gorham P.R. (1975). Toxicology and Pharmacological Action of *Anabaena flos-aquae* Toxin. Science.

[5] Thomas P., Stephens M., Wilkie G., Amar M., Lunt G.G., Whiting P., Gallagher T., Pereira E., Alkondon M., Albuquerque E.X. (1993). (+)-Anatoxin-a is a potent agonist at neuronal nicotinic acetylcholine receptors. J. Neurochem..

[6] Devlin J.P., Edwards O.E., Gorham P.R., Hunter N.R., Pike R.K., Stavric B. (1977). Anatoxin-a, a toxic alkaloid from *Anabaena flos-aquae* NRC-44h. Can. J. Chem..

[7] Zhong Y., Shen L., Ye X., Zhou D., He Y., Li Y., Ding Y., Zhu W., Ding J., Zhang H. (2020). Neurotoxic Anatoxin-a Can Also Exert Immunotoxicity by the Induction of Apoptosis on *Carassius auratus* Lymphocytes *in vitro* When Exposed to Environmentally Relevant Concentrations. Front. Physiol..

[8] Dittmann E., Wiegand C. (2006). Cyanobacterial toxins—Occurrence, biosynthesis and impact on human affairs. Mol. Nutr. Food Res..

[9] Carmichael W.W., Boyer G.L. (2016). Health impacts from cyanobacteria harmful algae blooms: Implications for the North American Great Lakes. Harmful Algae.

[10] Bownik A., Rymuszka A., Sierosławska A., Skowroński T. (2012). Anatoxin-a induces apoptosis of leukocytes and decreases the proliferative ability of lymphocytes of common carp (*Cyprinus carpio* L.) in vitro. Pol. J. Vet. Sci..

[11] Biré R., Bertin T., Dom I., Hort V., Schmitt C., Diogène J., Lemée R., De Haro L., Nicolas M. (2020). First Evidence of the Presence of Anatoxin-A in Sea Figs Associated with Human Food Poisonings in France. Mar. Drugs.

[12] Osswald J., Rellán S., Gago A., Vasconcelos V. (2008). Uptake and depuration of anatoxin-a by the mussel *Mytilus galloprovincialis* (Lamarck, 1819) under laboratory conditions. Chemosphere.

[13] Colas S., Duval C., Marie B. (2020). Toxicity, transfer and depuration of anatoxin-a (cyanobacterial neurotoxin) in medaka fish exposed by single-dose gavage. Aquat. Toxicol..

[14] Wiegand C., Pflugmacher S. (2005). Ecotoxicological effects of selected cyanobacterial secondary metabolites a short review. Toxicol. Appl. Pharmacol..

[15] Zhang C., Wang X., He J., Huang Y., Huang Q., Qin C., Qin J., Chen L. (2022). Neural excitotoxicity and the toxic mechanism induced by acute hypoxia in Chinese mitten crab (*Eriocheir sinensis*). Aquat. Toxicol..

[16] Chen Q., Luo Z., Liu X., Song Y., Liu C.X., Zheng J., Zhao Y. (2013). Effects of waterborne chronic copper exposure on hepatic lipid metabolism and metal-element composition in *Synechogobius hasta*. Arch. Environ. Contam. Toxicol..

[17] McIlwain D.R., Berger T., Mak T.W. (2013). Caspase functions in cell death and disease. Cold Spring Harb. Perspect. Biol..

[18] Didonna A., Sussman J., Benetti F., Legname G. (2012). The role of Bax and caspase-3 in doppel-induced apoptosis of cerebellar granule cells. Prion.

[19] Tufi S., Leonards P., Lamoree M., de Boer J., Legler J., Legradi J. (2016). Changes in Neurotransmitter Profiles during Early Zebrafish (*Danio rerio*) Development and after Pesticide Exposure. Environ. Sci. Technol..

[20] Aliakbarzadeh F., Rafiee M., Khodagholi F., Khorramizadeh M.R., Manouchehri H., Eslami A., Sayehmiri F., Mohseni-Bandpei A. (2023). Adverse effects of polystyrene nanoplastic and its binary mixtures with nonylphenol on zebrafish nervous system: From oxidative stress to impaired neurotransmitter system. Environ. Pollut..

[21] Osswald J., Rellán S., Gago A., Vasconcelos V. (2007). Toxicology and detection methods of the alkaloid neurotoxin produced by cyanobacteria, anatoxin-a. Environ. Int..

[22] Zhang R., Wei H., Xia Y., Du J. (2010). Development of light response and GABAergic excitation-to-inhibition switch in zebrafish retinal ganglion cells. J. Physiol..

[23] Wan P., Jin Q. (2017). Progress in research on the effects of dopamine and its receptor on the central nervous system. Med. J. Wuhan Univ..

[24] Cepeda C., Levine M.S. (2012). Dopamine-NMDA receptor interactions: Twenty years later. Dev. Neurosci..

[25] Kwon Y.H., Wang H., Denou E., Ghia J.E., Rossi L., Fontes M.E., Bernier S.P., Shajib M.S., Banskota S., Collins S.M. (2019). Modulation of Gut Microbiota Composition by Serotonin Signaling Influences Intestinal Immune Response and Susceptibility to Colitis. Cell. Mol. Gastroenterol. Hepatol..

[26] Fink K.B., Göthert M. (2007). 5-HT receptor regulation of neurotransmitter release. Pharmacol. Rev..

[27] Princivalle A.P. (2022). GABA_B_ Receptors in Neurodegeneration. Behav. Neurobiol. GABAB Recept. Funct..

[28] Yang Y., Yu Q., Zhang C., Wang X., He L., Huang Y., Li E., Qin J., Chen L. (2023). Acute thiamethoxam exposure induces hepatotoxicity and neurotoxicity in juvenile Chinese mitten crab (*Eriocheir sinensis*). Ecotoxicol. Environ. Saf..

[29] Feng H., Deng D., Zhu F., Chen S., Geng J., Jiang S., Zhang K., Jiang J., Yin S., Zhang C. (2025). Acute exposure to glufosinate-ammonium induces hepatopancreas toxicity in juvenile Chinese mitten crab (*Eriocheir sinensis*). J. Hazard. Mater..

[30] Chelyshev Y.A., Kabdesh I.M., Mukhamedshina Y.O. (2022). Extracellular Matrix in Neural Plasticity and Regeneration. Cell. Mol. Neurobiol..

[31] Ayub M., Jin H.-K., Bae J.-S. (2021). Novelty of Sphingolipids in the Central Nervous System Physiology and Disease: Focusing on the Sphingolipid Hypothesis of Neuroinflammation and Neurodegeneration. Int. J. Mol. Sci..

[32] Liao J., Cao Y., Zhao J., Yu B., Wang Y., Li W., Li H., Lv S., Wen W., Cui H. (2023). Aqueous extract of *Polygala japonica* Houtt. ameliorated nonalcoholic steatohepatitis in mice through restoring the gut microbiota disorders and affecting the metabolites in feces and liver. Phytomedicine.

[33] Panther E.J., Dodd W., Clark A., Lucke-Wold B. (2022). Gastrointestinal Microbiome and Neurologic Injury. Biomedicines.

[34] Huo W., Qiao Y., Li E., Li M., Che L. (2025). Interplay between nutrition, microbiota, and immunity in rotavirus infection: Insights from human and animal models. Front. Vet. Sci..

[35] Shao R., Tan X., Pan M., Huang J., Huang L., Bi B., Huang X., Wang J., Li X. (2024). Inulin alters gut microbiota to alleviate post-stroke depressive-like behavior associated with the IGF-1-mediated MAPK signaling pathway. Brain Behav..

[36] Bravo J.A., Forsythe P., Chew M.V., Escaravage E., Savignac H.M., Dinan T.G., Bienenstock J., Cryan J.F. (2011). Ingestion of *Lactobacillus* strain regulates emotional behavior and central GABA receptor expression in a mouse via the vagus nerve. Proc. Natl. Acad. Sci. USA.

[37] Kulinich A., Wang Q., Duan X.C., Lyu Y.M., Zhang X.Y., Awad F.N., Liu L., Voglmeir J. (2020). Biochemical characterization of the endo-α-N-acetylgalactosaminidase pool of the human gut symbiont *Tyzzerella nexilis*. Carbohydr. Res..

[38] Boonchooduang N., Louthrenoo O., Likhitweerawong N., Kunasol C., Thonusin C., Sriwichaiin S., Nawara W., Chattipakorn N., Chattipakorn S.C. (2025). Impact of psychostimulants on microbiota and short-chain fatty acids alterations in children with attention-deficit/hyperactivity disorder. Sci. Rep..

[39] Takewaki D., Kiguchi Y., Masuoka H., Manu M.S., Raveney B.J.E., Narushima S., Kurokawa R., Ogata Y., Hattori M., Kimura Y. (2025). *Tyzzerella nexilis* strains enriched in mobile genetic elements are involved in progressive multiple sclerosis. Cell Rep..

[40] Abu Y.F., Singh S., Tao J., Chupikova I., Singh P., Meng J., Roy S. (2024). Opioid-induced dysbiosis of maternal gut microbiota during gestation alters offspring gut microbiota and pain sensitivity. Gut Microbes.

[41] Feng H., Song L., Wu Y., Zhao F., Zhu F., Song Z., Zhang K., Jiang J., Cai X., Yin S. (2025). Novel insight into the mechanisms of neurotoxicity induced by glufosinate-ammonium via the microbiota-intestine-brain axis in Chinese mitten crab (*Eriocheir sinensis*). Pestic. Biochem. Physiol..

