# Supplementary material for: Neurotoxicity and Intestinal Microbiota Dysbiosis in the Chinese Mitten Crab (Eriocheir sinensis) Induced by Anatoxin-a: A Microbiota–Intestine–Brain Axis Perspective
Source: Microorganisms. 2025 Oct 15;13(10):2380. doi: 10.3390/microorganisms13102380 (PMC12565913; doi:10.3390/microorganisms13102380)
Supplement: Supplementary file 1 [file microorganisms-13-02380-s001.zip › Figure S1 and S2.pdf]

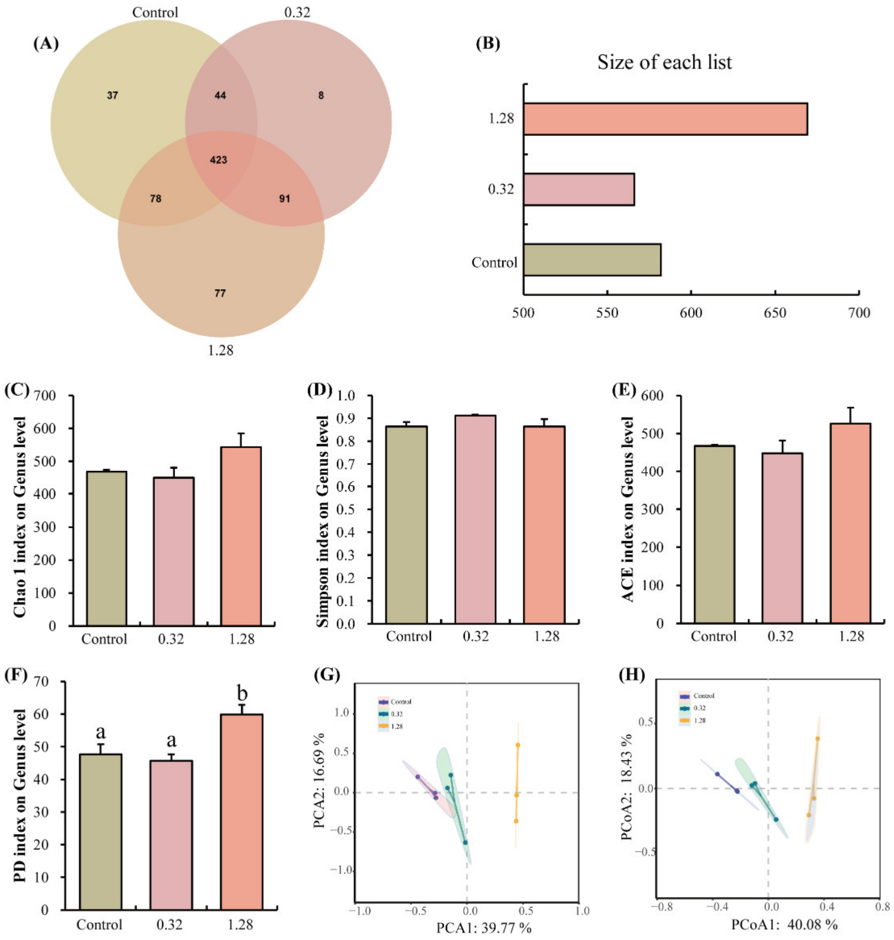

Figure S1. The diversity of intestinal microbial in different groups of the juvenile *E. sinensis*. (A) Venn diagram showing the specific and shared OTUs in each group; (B) Histogram showing each group of OTUs; (C) Chao indexes; (D) Simpson indexes; (E) ACE indexes; (F) PD indexes; (G) Beta diversity indicated by PCA; (H) Beta diversity indicated by PCoA.

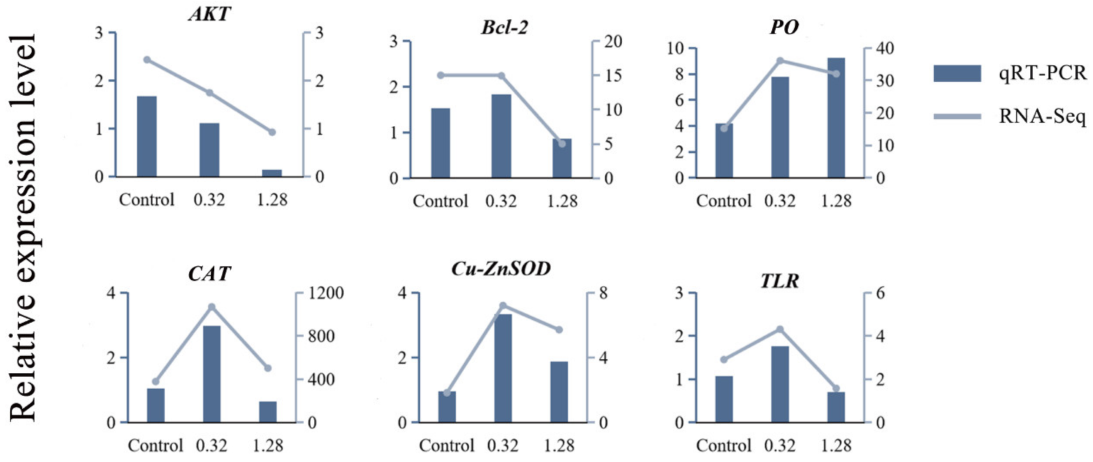

Figure S2. Validation of gene expression patterns by qPCR.
