# Supplementary material for: Neurotoxicity and Intestinal Microbiota Dysbiosis in the Chinese Mitten Crab (Eriocheir sinensis) Induced by Anatoxin-a: A Microbiota–Intestine–Brain Axis Perspective
Source: Microorganisms. 2025 Oct 15;13(10):2380. doi: 10.3390/microorganisms13102380 (PMC12565913; doi:10.3390/microorganisms13102380)
Supplement: Supplementary file 1 [file microorganisms-13-02380-s001.zip › Table S1 and S2.pdf]

Table S1. Primer information for quantitative real-time polymerase chain reaction

Primers	Forward Sequences (5'-3')	Reverse Sequences (5'-3')
Bax	AGAGATGAAGCAGACCACGC	TTCTACGGTGGGTGAGTCCA
Bcl 2	CATCATCTCCCTCTTCGCGG	CAGTCCCATCACGTCGATCA
Caspase 3	AGTTTTGGGGAGGTGC	TCAGGTTCCGTGGGTT
Caspase 8	CATGGTGATGAGAATGAC	TTGGATGAAGTAGAGACG
P53	ATGTGCCTTGGCTCCAGTGTTG	TCGTCAGTCTTGATGTCTCGTGTG
NMDA 1AR	TATGGTTTGCCTGGGGAGTA	CAGGTTGGCAGTGTATGAGG
NMDA 2AR	GGTGTCCCCTCACGTATTTG	AAGCAGAACAAGAGAGCGTC
NMDA 2BR	TGGAGACACTCTGCGACAAA	AGGTCTCCGAGTTGAACAGG
GABA 2BR	GTTGCATTATGACCGACCTCC	ACGCCATCATACGTGTAGCC
5-HT 1BR	GCCAGGAAGCGCATCAGACGCA	GGGTGAGGGCTGAGAGGACAT
5-HT 2BR	AGGCGACGAAGGTTCTGGGTGTGGT	ACCAGGTTGATCATCTCCTCCCCGA
5-HT 7R	ATCATTATGAGCGCCTTCGT	AGGCACAGAGTCTCCTGGAA
DA 1AR	CCGGACAGCTCCACCAAAGT	AGGGCAGCCAGCACACGATA
DA 2AR	TGCTATTATCTGGGTGGTGT	ATGATGAAGTCTGCGTTGTG
β-actin	TCATCACCATCGGCAATGA	TTGTAAGTGGTCTCGTGGATG
S27	GGTCGATGACAATGGCAAGA	CCACAGTACTGGCGGTCAAA

Table S2. Evaluation of RNA-Seq Data

Samples	Raw reads	Raw bases	Clean reads	Clean bases	Q20(%)	Q30(%)	GC content(%)
A1-1	46600432	6990065000	46576834	6961609000	97.08	93.02	42.89
A1-2	45401110	6609068000	45377190	6779071000	97.08	93.06	43.30
A1-3	52619996	7892999000	52606626	7863387000	97.48	93.74	40.41
A1-4	48102524	7215379000	48091178	7180007000	97.33	93.55	40.10
A1-5	47680610	7152092000	47656232	7119511000	97.34	93.65	36.55
A2-1	48991020	7348653000	48951890	7314570000	96.27	91.41	42.86
A2-2	43488050	6523208000	43445282	6491028000	95.65	90.33	42.02
A2-3	54290328	8143549000	54244104	8102904000	95.56	90.14	42.00
A2-4	64091352	9613703000	64053910	9567871000	95.79	90.49	44.99
A3-1	48286986	7243048000	48242250	7204144000	95.63	90.31	42.54
A3-2	40410760	6061614000	40367204	6025526000	95.62	90.34	44.30
A3-3	45643618	6846543000	45597780	6799794000	96.15	91.40	38.53
A3-4	43328310	6499246000	43271042	6451097000	96.76	92.89	38.15
