# Supplementary material for: Neurotoxicity and Intestinal Microbiota Dysbiosis in the Chinese Mitten Crab (Eriocheir sinensis) Induced by Anatoxin-a: A Microbiota–Intestine–Brain Axis Perspective
Source: Microorganisms. 2025 Oct 15;13(10):2380. doi: 10.3390/microorganisms13102380 (PMC12565913; doi:10.3390/microorganisms13102380)
Supplement: Supplementary file 1 [file microorganisms-13-02380-s001.zip › Details of intestinal microbiome analysis.pdf]

## Analysis of the intestinal microbiome

### 1. DNA extraction and PCR amplification

Total microbial genomic DNA was extracted from intestinal samples using the E.Z.N.A.® DNA Kit (Omega Bio-tek, Norcross, GA, U.S.) according to manufacturer's instructions. The quality and concentration of DNA were determined by 1.0% agarose gel electrophoresis and a NanoDrop2000 spectrophotometer (Thermo Scientific, United States) and kept at -80 ℃ prior to further use. The hypervariable region V3-V4 of the bacterial 16S rRNA gene were amplified with primer pairs 338F (5'-ACTCCTACGGGAGGCAGCAG-3') and 806R (5'-GGACTACHVGGGTWTCTAAT-3')[1] byT100 Thermal Cycler PCR thermocycler (BIO-RAD, USA). The PCR reaction mixture including 10μL 2×Pro Taq, 0.8 μL Forward Primer (5 μM) and 0.8μL Reverse Primer (5 μM), 10ng of Template DNA, and $ddH_2O$ to a final volume of 20 μL. PCR amplification cycling conditions were as follows: initial denaturation at 95 ℃ for 3 min, followed by 27 cycles of denaturing at 95 ℃ for 30 s, annealing at 55 ℃ for 30 s and extension at 72 ℃for 45 s, and single extension at 72 ℃ for 10 min, and end at 4 ℃. The PCR product was extracted from 2% agarose gel and purified using the PCR Clean-Up Kit (YuHua, Shanghai, China) according to manufacturer's instructions and quantified using Qubit 4.0 (Thermo Fisher Scientific, USA).

### 2. Illumina sequencing

Purified amplicons were pooled in equimolar amounts and paired-end sequenced on an Illumina Nextseq2000 platform (Illumina, San Diego,USA) according to the standard protocols by Majorbio Bio-Pharm Technology Co. Ltd. (Shanghai, China).

### 3. Amplicon sequence processing and analysis

After demultiplexing, the resulting sequences were quality filtered with fastp (0.19.6)[3] and merged with FLASH ( v1.2.11)[2]. Then the high-quality sequences were de-noised using DADA2/ Deblur [4, 5] plugin in the Qiime2[6] (version 2020.2) pipeline with recommended parameters, which obtains single nucleotide resolution based on error profiles within samples. DADA2/ Deblur denoised sequences are usually called amplicon sequence variants (ASVs). To minimize the effects of sequencing depth on alpha and beta diversity measure, the number of sequence from each sample was rarefied to 20,000, which still yielded an average Good's coverage of 97.90%.

Taxonomic assignment of ASVs was performed using the Naive bayes (or Vsearch/Blast) consensus taxonomy classifier implemented in Qiime2 and the SILVA 16S rRNA database (v138). The metagenomic function was predicted by PICRUSt2 (Phylogenetic Investigation of Communities by Reconstruction of Unobserved States) [7] based on ASV representative sequences. PICRUSt2 is a software containing a series of tools as follows: HMMER was used to aligns ASV representative sequences with reference sequences. EPA-NG and Gappa were used to put ASV representative sequences into a reference tree. The castor was used to normalize the 16S gene copies. MinPath was used to predict gene family profiles, and locate into the gene pathways. Entire analysis process was accord to protocols of PICRUSt2.

**4. Statistical Analysis**

Based on the ASVs information, rarefaction curves and alpha diversity indices including observed ASVs, Chao1 richness, Shannon index and Good's coverage were calculated with Mothur v1.30.1[8]. The similarity among the microbial communities in different samples was determined by principal coordinate analysis (PCoA) based on Bray-curtis dissimilarity using Vegan v2.5-3 package. The PERMANOVA test was used to assess the percentage of variation explained by the treatment along with its statistical significance using Vegan v2.5-3 package. The linear discriminant analysis (LDA) effect size (LEfSe)[9] (http://huttenhower.sph.harvard.edu/LEfSe) was performed to identify the significantly abundant taxa (phylum to genera) of bacteria among the different groups (LDA score > 2, $P < 0.05$). The variance inflation factor (VIF) for each variable was estimated using the vif function in the car package (https://cran.r-project.org/web/packages/car/car.pdf). The distance-based redundancy analysis (db-RDA) was performed using Vegan v2.5-3 package to investigate intestinal bacterial community structure. Forward selection was based on Monte Carlo permutation tests (permutations = 9999). The co-occurrence networks were constructed to explore the internal community relationships across the samples[10]. A correlation between two nodes was considered to be statistically robust if the spearman's correlation coefficient over 0.6 or less than -0.6, and the *P*-value less than 0.01.

## References


[1] Liu C, Zhao D, Ma W, et al. Denitrifying sulfide removal process on high-salinity wastewaters in the presence of Halomonas sp[J]. Applied microbiology and biotechnology, 2016, 100(3): 1421-1426. doi:10.1007/s00253-015-7039-6

[2]Chen S, Zhou Y, Chen Y, et al. fastp: an ultra-fast all-in-one FASTQ preprocessor[J]. Bioinformatics, 2018, 34(17): i884-i890. doi:10.1093/bioinformatics/bty560

[3] Tanja, Mago, Steven, et al. FLASH: fast length adjustment of short reads to improve genome assemblies[J]. Bioinformatics, 2011, 27(21):2957-2963. doi:10.1093/bioinformatics/btr507

[4] Edgar, Robert C. UPARSE: highly accurate ASV sequences from microbial amplicon reads[J]. Nature Methods, 2013, 10(10):996-998. doi:10.1038/nmeth.2604

[5] Stackebrandt E, Goebel B M. Taxonomic Note: A Place for DNA-DNA Reassociation and 16S rRNA Sequence Analysis in the Present Species Definition in Bacteriology[J]. Int. J. Syst. Bacteriol, 1994, 44(4):846-849. doi:10.1099/00207713-44-4-846

[6] Wang Q. Naive Bayesian classifier for rapid assignment of rRNA sequences into the new bacterial taxonomy[J]. Appl. Environ. Microbiol, 2007, 73. doi:10.1128/AEM.00062-07

[7] Douglas G M, Maffei V J, Zaneveld J R, et al. PICRUSt2 for prediction of metagenome functions[J]. Nature Biotechnology. 2020, 38: 685 – 688. doi:10.1038/s41587-020-0548-6

[8] Schloss P D, Westcott S L, Ryabin T, et al. Introducing mothur: Open-Source, Platform-Independent, Community-Supported Software for Describing and Comparing Microbial Communities[J]. Applied & Environmental Microbiology. 2009, 75:7537. doi:10.1128/AEM.01541-09

[9] Segata N, Izard J, Waldron L, et al. Metagenomic biomarker discovery and explanation[J]. Genome Biology, 2011, 12: R60. doi:10.1186/gb-2011-12-6-r60

[10] Barberan A, Bates ST, Casamayor EO, Fierer N. Using network analysis to explore co-occurrence patterns in soil microbial communities[J]. The ISME Journal, 2012, 6:343-351. doi:10.1038/ismej.2011.119
